# ﻿Review of the millipede genus *Orthomorpha* Bollman, 1893 (Diplopoda, Polydesmida, Paradoxosomatidae) in Cambodia, with new records and descriptions of three new species

**DOI:** 10.3897/zookeys.1251.158776

**Published:** 2025-09-10

**Authors:** Natdanai Likhitrakarn, Sergei I. Golovatch, Sothearen Thi, Chhin Sophea, Vanny Lou, Pablo Sinovas, Ekgachai Jeratthitikul, Arthit Pholyotha, Warut Siriwut, Ruttapon Srisonchai, Somsak Panha, Chirasak Sutcharit

**Affiliations:** 1 Program of Agriculture, Faculty of Agricultural Production, Maejo University, Chiang Mai 50290, Thailand Department of Biodiversity, General Directorate of Policy and Strategy, Ministry of Environment Chiang Mai Thailand; 2 Institute of Ecology and Evolution, Russian Academy of Sciences, Leninsky pr. 33, Moscow, 119071, Russia Institute of Ecology and Evolution, Russian Academy of Sciences, Moscow Russia; 3 Fauna & Flora Cambodia Programme, 19 Street 360, Phnom Penh, Cambodia Fauna & Flora Cambodia Programme Phnom Penh Cambodia; 4 Department of Biodiversity, General Directorate of Policy and Strategy, Ministry of Environment, Phnom Penh, Cambodia Ministry of Environment Phnom Penh Cambodia; 5 Animal Systematics and Molecular Ecology Laboratory, Department of Biology, Faculty of Science, Mahidol University, Bangkok 10400, Thailand Animal Systematics and Molecular Ecology Laboratory, Department of Biology, Faculty of Science, Mahidol University Bangkok Thailand; 6 Animal Systematics Research Unit, Department of Biology, Faculty of Science, Chulalongkorn University, Bangkok 10330, Thailand Animal Systematics Research Unit, Department of Biology, Faculty of Science, Chulalongkorn University Bangkok Thailand; 7 Department of Biology, Faculty of Science, Khon Kaen University, Khon Kaen 40002, Thailand Animal Systematics Research, Department of Biology, Faculty of Science, Khon Kaen University Khon Kaen Thailand; 8 Academy of Science, The Royal Society of Thailand, Bangkok 10300, Thailand Academy of Science, The Royal Society of Thailand Bangkok Thailand

**Keywords:** *
Asiomorpha
*, catalogue, distribution, key, morphology, Orthomorphini, taxonomy

## Abstract

The Southeast Asian millipede genus *Orthomorpha* Bollman, 1893 currently encompasses 59 accepted species, including three newly described from Cambodia: *Orthomorpha tergoaurantia* Likhitrakarn, **sp. nov.**, *Orthomorpha efefai* Likhitrakarn, **sp. nov.**, and *Orthomorpha battambangiensis* Likhitrakarn, **sp. nov.** The new species are described based on distinctive morphological traits, with particular emphasis on gonopodal features. Species descriptions are accompanied by detailed illustrations and scanning electron micrographs (SEM) of the gonopods. An updated identification key to the known *Orthomorpha* species from Cambodia is also provided.

## ﻿Introduction

The Southeast Asian millipede genus *Orthomorpha* Bollman, 1893, is among the largest in the tribe Orthomorphini, family Paradoxosomatidae, previously comprising 56 accepted species (Likhitrakarn et al. 2011, 2014a, 2019). The present contribution adds another three species, bringing the total to 59. The genus is widespread throughout Southeast Asia, extending from northern Myanmar and southern China in the northwest to Lombok Island in Indonesia in the southeast (Likhitrakarn et al. 2011). Only a single species, *Orthomorpha coarctata* (de Saussure, 1860), often also assigned to the genus *Asiomorpha* Verhoeff, 1939, shows a pantropical distribution as a result of human activities (Shelley and Lehtinen 1998; Likhitrakarn et al. 2011, 2019, 2023; Nguyen and Sierwald 2013).

Recent taxonomic studies on *Orthomorpha* have mainly been based on traditional morphological characters, with emphasis on gonopodal structures known to be of great taxonomic importance in most Diplopoda. These studies have revealed this genus to be highly species-rich and common, particularly in Thailand, Laos, and Vietnam (Golovatch 1997; Likhitrakarn et al. 2010, 2011, 2014a, 2019).

The millipede fauna of Cambodia is among the least explored globally, and is considerably understudied compared to those of other Southeast Asian countries. The genus *Orthomorpha* has, until now, been represented in Cambodia by three species: *O. coarctata* (de Saussure, 1860), *O. hydrobiologica* Attems, 1930, and *O. cambodjana* (Attems, 1953) (Likhitrakarn et al. 2015).

Recent field studies in Cambodia have revealed some new records and three new species of *Orthomorpha*. The present paper provides their morphological diagnoses and descriptions. In addition, we provide a taxonomic key to all six species of the genus currently documented from Cambodia, with their distribution map.

## ﻿Material and methods

Millipede specimens were manually collected from various localities in Cambodia during 2019–2024. All specimens were handled and euthanized following ethical guidelines outlined by the American Veterinary Medical Association (AVMA 2020). Live samples were photographed in the laboratory using a Nikon 700D digital camera equipped with a Nikon AF-S VR 105 mm macro lens. The entire protocol received approval from the Chulalongkorn University Animal Care and Use Committee (Protocol Review No. 1723018). Following euthanasia, the millipedes were preserved in 75% ethanol for further morphological examination. Morphological characters were studied, measured, and photographed using a Nikon SMZ 745T trinocular stereo microscope coupled with a Canon EOS 5DS R digital SLR camera. Digital images were processed and enhanced using Adobe Photoshop CS6. Line drawings were executed from photographs and further checked using a stereo microscope fitted with a digital SLR camera. For scanning electron microscopy (SEM), the gonopods were coated with an 8 nm gold layer utilizing a CCU-010 high vacuum sputter and carbon coater (Safematic). SEM images were obtained with a TESCAN VEGA3 scanning electron microscope operating at 5 keV acceleration voltage. After SEM examination, gonopods were returned to ethanol with the gold coating intact.

All type material is housed in the Museum of Zoology, Chulalongkorn University (**CUMZ**), Bangkok, Thailand.

The terminology for denoting the gonopodal and somatic structures primarily follows Likhitrakarn et al. (2010, 2011, 2014a, 2019). The abbreviations used for specific gonopodal structures are as follows: **cx** = coxite, **fe** = femoral part, **pfe** = prefemoral part, **sl** = solenomere, and **sph** = solenophore.

Coordinates and elevations were documented by Garmin GPSMAP 60 CSx and Garmin eTrex 30 devices with the WGS84 datum. Subsequently, Google Earth Pro ver. 7.3.6 was implemented to confirm the precision of the recorded data.

In the catalogue sections, D represents the original description or subsequent descriptive notes; L for the appearance in a species list; R refers to a subsequent record or records, K to appearance in a key, and M to merely mentioning.

## ﻿Taxonomy

**Class Diplopoda de Blainville in Gervais, 1844**

**Order Polydesmida Leach, 1815**

**Family Paradoxosomatidae Daday, 1889**

**Tribe Orthomorphini Brölemann, 1916**

### ﻿Genus Orthomorpha

Taxon classificationAnimaliaPolydesmidaParadoxosomatidae

Bollman, 1893

E13CA985-9D2C-5202-B2A4-49FD7960524B

Orthomorpha coarctata (de Saussure, 1860)
Polydesmus coarctatus de Saussure, 1860: 297 (D).
Paradesmus flavocarinatus Daday, 1889: 136 (D). Synonymized by Enghoff (2005).
Orthomorpha coarctata – Pocock 1895: 809 (R, M, K); Attems 1937: 62 (D); 1953: 179 (R); Jeekel 1968: 45 (M); Likhitrakarn et al. 2011: 12 (D, R, K), Golovatch and Wesener 2016: 47 (L).
Orthomorpha coarctata gigas Attems, 1927: 63 (D). Synonymized by Jeekel (1968).
Asiomorpha coarctata – Verhoeff 1939: 117 (D); Enghoff 2005: 95, 96 (R); Nguyen and Sierwald 2013: 1236 (L); Likhitrakarn et al. 2023: 71 (R); et auctorum.
Orthomorpha coarctata gigas – Jeekel 1968: 45 (M); Golovatch 1998: 43 (K).

#### New material examined.

4 ♂, 13 ♀ (SMF-011), Cambodia, Battambang Province, Sangker River, walls and vegetation along river, 25 m a.s.l., 13°06'6.45"N, 103°11'59.09"E, 18.07.2017, leg. P. Jäger.

#### Records from Cambodia.

Koh Kong Province, Sre Ambel (Attems 1953).

#### Remarks.

*Orthomorpha coarctata* (de Saussure, 1860) is a pantropical anthropochore species which is often referred to the monotypic genus *Asiomorpha* Verhoeff, 1939. However, in line with the comprehensive revision of the genus *Orthomorpha* by Likhitrakarn et al. (2011), we retain this species within *Orthomorpha*. This decision follows the reasoning of Jeekel (1968), who argued that the characters separating *Asiomorpha* from *Orthomorpha* (primarily the gonopod tip which is reduced to a single, simple lobe) are insufficient to warrant a distinct generic status when considering the full range of variation within *Orthomorphasensu lato.*

Since some recent checklists, including one by the part of the present authors (Likhitrakarn et al. 2023) and the global catalogue of Paradoxosomatidae (Nguyen and Sierwald 2013), have applied the generic name *Asiomorpha*, further studies have been using the name for consistency with these particular reference works. Further complicating this matter, the molecular analysis by Likhitrakarn et al. (2019) demonstrated that *O. coarctata* forms a distinct clade, genetically distant from other congeners. This pronounced genetic isolation renders the genus *Orthomorpha* polyphyletic, unless *O. coarctata* is treated within its own monotypic genus, *Asiomorpha*. However, the authors of that study themselves urged caution, noting that their phylogram was based on a single mitochondrial gene (COI) and should thus be regarded as provisional. To definitively resolve the phylogenetic relationships within the Orthomorphini and reveal the taxonomic status of *Asiomorpha*, future studies incorporating additional genetic markers are clearly warranted.

The present contribution, being a focused taxonomic review of the genus *Orthomorpha* in a specific region, requires a consistent generic concept. Therefore, our treatment here is based on the taxonomic framework established in the most recent revision of the genus (Likhitrakarn et al. 2011), which includes the full taxonomic history of *O. coarctata*.

### ﻿ Orthomorpha hydrobiologica

Taxon classificationAnimaliaPolydesmidaParadoxosomatidae

Attems, 1930

3C0337D2-D417-5D45-BA5D-F98B095F238E

Orthomorpha hydrobiologica Attems, 1930: 120 (D).
Orthomorpha hydrobiologica – Attems 1937: 63 (D); 1938: 215 (R); Jeekel 1963: 265 (M); 1964: 361 (M, D); 1968: 45 (M); Hoffman 1973: 362 (M); 1977: 700 (M); Golovatch 1998: 42 (M); Enghoff et al. 2004: 38 (R); Likhitrakarn et al. 2011: 53 (D); 2015: 181 (R); Likhitrakarn et al. 2019: 127 (L); Nguyen et al. 2025: 48 (L).
Oxidus hydrobiologicus – Chamberlin 1945: 10 (R).

#### Records from Cambodia.

Sihanoukville Province, Ream; Kampot Province, Phnom Bokor (Attems 1938). Also known from Indonesia (Attems 1930; Chamberlin 1945) and Vietnam (Attems 1938; Enghoff et al. 2004; Likhitrakarn et al. 2019).

#### Remarks.

This species has been redescribed relatively recently, based on type material (Likhitrakarn et al. 2011). It demonstrates a broad coastal distribution pattern along the South China Sea, ranging from northern Vietnam to southern Cambodia, with its extensive presence presumably due to human-mediated dispersal (Likhitrakarn et al. 2019).

### ﻿ Orthomorpha cambodjana

Taxon classificationAnimaliaPolydesmidaParadoxosomatidae

(Attems, 1953)

8A07A8F5-9A1C-5AD2-AEC4-94C95BEDF0F8

Pratinus cambodjanus Attems, 1953: 168 (D).
Orthomorpha cambodjana – Jeekel 1963: 265 (M), 1964: 361 (M, D), 1968: 56 (M); Hoffman 1977: 700 (M); Golovatch 1998: 42 (M, D); Likhitrakarn et al. 2011: 66 (D), 2014a: 7 (D, R).

#### Records from Cambodia.

Kampot Province, Kampot; Sihanoukville Province, Ream; Koh Kong Province, Sre Ambel (Attems 1953). Also known from Laos (Likhitrakarn et al. 2014a).

#### Remarks.

This species has been redescribed relatively recently (Likhitrakarn et al. 2011), based on type material. That redescription, coupled with subsequent records, suggests a broader distribution of the species in Indochina than previously recognized (Likhitrakarn et al. 2011, 2014a).

### ﻿ Orthomorpha tergoaurantia

Taxon classificationAnimaliaPolydesmidaParadoxosomatidae

Likhitrakarn sp. nov.

DFFD7DF8-8710-59E8-9737-88FFE7523BEA

https://zoobank.org/6E76D3DC-E0BF-46C4-9F68-4E335DAB1B2C

Figs 1, 2, 3, 4

#### Material examined.

***Holotype***: ♂ (CUMZ-PD0031), Cambodia, Kampot Province, Banteay Meas District, Prasat Phnom Totong Temple (locality code C042), ca 50 m a.s.l., 10°41'50"N, 104°31'21"E, 15.09.2019, leg. R. Srisonchai. ***Paratypes***: 2 ♀♀ (CUMZ-PD0031), same data, together with holotype. 1 ♂, 1 ♀ (CUMZ-PD0032), Cambodia, Kampot Province, Tuek Chhou District, Phnom Chhngok Cave Temple (locality code C046), ca 60 m a.s.l., 10°38'35"N, 104°16'04"E, 16.09.2019, leg. R. Srisonchai; 2 ♂♂, 1 ♀ (CUMZ-PD0033), Cambodia, Kampong Speu Province, Samraong Tong District, Khum Skuh, Phnom Cheal Pagoda (locality code C031), ca 210 m a.s.l., 11°23'12"N, 104°30'35"E, 13.09.2019, leg. R. Srisonchai; 3 ♂♂ (CUMZ-PD034), Cambodia, Kampot Province, Krong Kampot District, side of Road no. 33, beside the Preaek Tuek Chhu River (locality code C049), ca 5 m a.s.l., 10°36'41.2"N, 104°13'23.8"E, 16.09.2019, leg. R. Srisonchai.

#### Etymology.

The species name ‘tergoaurantia’ is derived from the Latin words ‘tergum’, meaning ‘back’, and ‘aurantium’, meaning ‘orange’. This name emphasizes the distinctive dark or bright orange coloration of the paraterga, which is a prominent characteristic distinguishing it from other Cambodian congeners.

#### Diagnosis.

This new species seems to be particularly similar to *Orthomorpha cambodjana* (Attems, 1953) in gonopod conformation, sharing a very slender and suberect gonopodal telopodite. However, it clearly differs by its larger body size (35.4–39.4 mm long and 4.1–4.9 mm wide in ♂, 35.4–39.4 mm long and 4.2–4.3 mm wide in ♀, *vs* 17–30 mm long and 2.0–3.1 mm wide in ♂, 19–29 mm long and 2.0–3.4 mm wide in ♀ of *O. cambodjana*) and by the shape of the solenophore apex. The caudal denticle on the pleurosternal carinae is traceable until body rings 7 or 8 in the new species (*vs* rings 16 or 17 in *O. cambodjana*). Additionally, tarsal brushes are present until ♂ legs of ring 17 in the new species, *vs* only until ♂ legs 7 of *O. cambodjana*.

#### Description.

Length 31.2–37.3 mm (♂), 35.4–39.4 mm (♀), width of midbody pro- and metazona 2.5–3.2 and 4.1–4.9 mm (♂) or 3.2–3.9 and 4.7–5.7 mm (♀), respectively.

**Figure 1.**
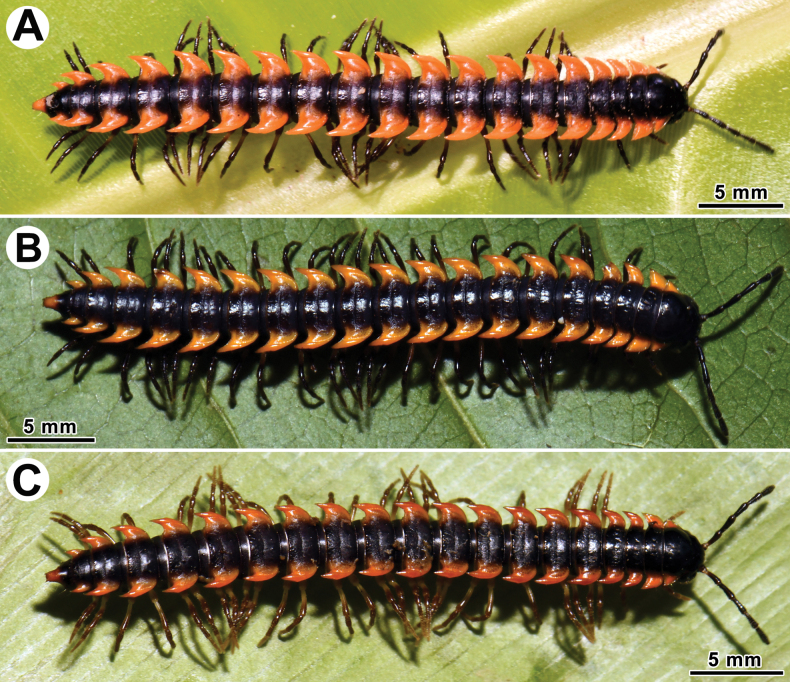
*Orthomorpha tergoaurantia* Likhitrakarn, sp. nov., habitus, live coloration. A. ♂ Paratype from Phnom Cheal Pagoda; B. ♀ Paratype from Phnom Chhngok Cave Temple; C. ♂ holotype from Prasat Phnom Totong Temple.

**Figure 2.**
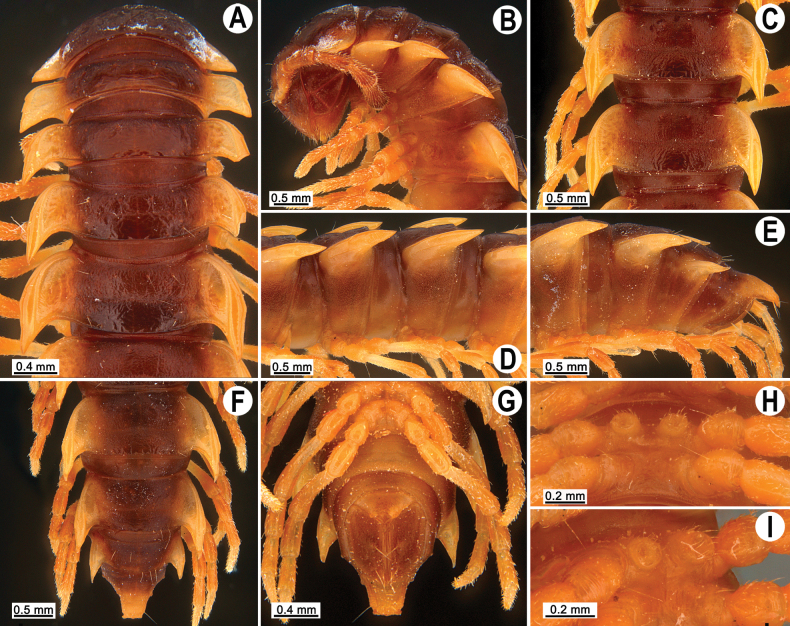
*Orthomorpha tergoaurantia* Likhitrakarn, sp. nov., ♂ holotype. A, B. Anterior part of body, dorsal and lateral views, respectively; C. Rings 10 and 11, dorsal view; D. Rings 9–11, lateral view; E–G. Posterior part of body, lateral, dorsal and ventral views, respectively; H, I. Sternal cones between coxae 4, subcaudal and sublateral views, respectively.

Coloration of live animals dark brown to blackish (Fig. 1), with contrasting dark orange to bright orange or yellowish paraterga and epiproct; antennae black; venter and legs brown to blackish; coloration of alcohol material after six years of preservation faded to dark castaneous brown, paraterga, venter, epiproct, and several basal podomeres more flavous, pale pinkish, brownish or pale yellow (Fig. 2A–G).

Clypeolabral region and vertex sparsely setose, epicranial suture distinct. Antennae long (Fig. 2B), reaching or extending past ring 3 (♂) or reaching to ring 3 (♀) when stretched dorsally. In width, head < collum < ring 3 < 4 < 2 < 5 < 6 < 7–16 (Fig. 2A), thereafter body gently and gradually tapering. Collum with three transverse rows of strong setae: 4+4 anterior, 2+2 intermediate, and 4+4 posterior; a small incision laterally in posterior 1/3; caudal corner of paraterga pointed, dentiform, paraterga declined ventrad, not drawn past rear margin (Fig. 2A, B).

Tegument smooth and shining, prozona finely shagreened, metaterga smooth and leathery, posterior halves often rugulose, surface below paraterga microgranulate (Fig. 2A–F). Postcollum metaterga with two transverse rows of rather long setae: 2+2 in anterior and 3+3 in posterior row (Fig. 2A, C, F), the latter also borne on wrinkles and often abraded, but then readily traceable as insertion points. Tergal setae long, strong, slender, about 1/3 metatergal length (Fig. 2A, D–F). Axial line visible both on pro- and metazona. Paraterga very strongly developed (Fig. 2A–F), set high (at ca 1/4 metazonal height), upturned in ♂, lying below dorsum on rings 2–5 and 17–19, above dorsum on rings 6–16, in ♀ mostly below dorsum, rather thin in lateral view, a little thicker on pore-bearing rings (Fig. 2B, D, E); anterior margin well-developed, mostly regularly rounded and narrowly bordered, fused to callus; caudal corner narrowly rounded, on postcollum rings extending increasingly past rear tergal margin, better so in ♂, nearly pointed to pointed, caudal tip on paraterga 16–19 evidently curved mesad (Fig. 2E, F). Posterior margin of paraterga clearly concave, especially so in rings 15–19. Calluses on paraterga delimited by a sulcus only dorsally. Paraterga 2 broad, lateral margin with three small incisions, the one near caudal corner being particularly small (Fig. 2A). Paraterga 3 and 4 with two small incisions at lateral margin, one at midway, the other at posterior 1/3; anterior incision particularly evident. Lateral margins of following paraterga often with a setigerous incision in anterior 1/3, being smaller on pore-bearing rings (Fig. 2C). Ozopores evident, lateral, lying in an ovoid groove at about 1/3 metatergal length in front of posterior margin of metaterga (Fig. 2B, D, E). Transverse sulcus usually distinct (Fig. 2A, C, F), slightly incomplete on ring 4, complete and clearly visible on metaterga 5–18, narrow, rather deep, reaching the bases of paraterga, arcuate, beaded at bottom. Stricture between pro- and metazona narrow, deep, beaded at bottom down to base of paraterga (Fig. 2A, C, F). Pleurosternal carinae complete crests on rings 2 and 3, a sharp caudal tooth on ring 4, the tooth gradually reduced into small, caudally roughly granulate crests until ring 7(8), thereafter missing (♂, ♀) (Fig. 2B, D).

Epiproct (Fig. 2E–G) conical, flattened dorsoventrally, subtruncate, with two evident apical papillae directed ventrocaudally (Fig. 2E, G); pre-apical papillae small, but evident, lying close to tip. Hypoproct subtriangular (Fig. 2G), 1+1 setiferous knobs at caudal edge well-separated and evident.

Sterna sparsely setose, without modifications; cross-impressions rather deep; a paramedian pair of evident, rounded, fully separated, setose cones between ♂ coxae 4 (Fig. 2H, I). A conspicuous ridge in front of gonopod aperture. Legs long and slender, midbody ones ca 1.3–1.5 (♂) (Fig. 2B, F, G) or 1.1–1.3 times (♀) as long as body height, prefemora without modifications, ♂ tarsal brushes present until legs of ring 17.

**Figure 3.**
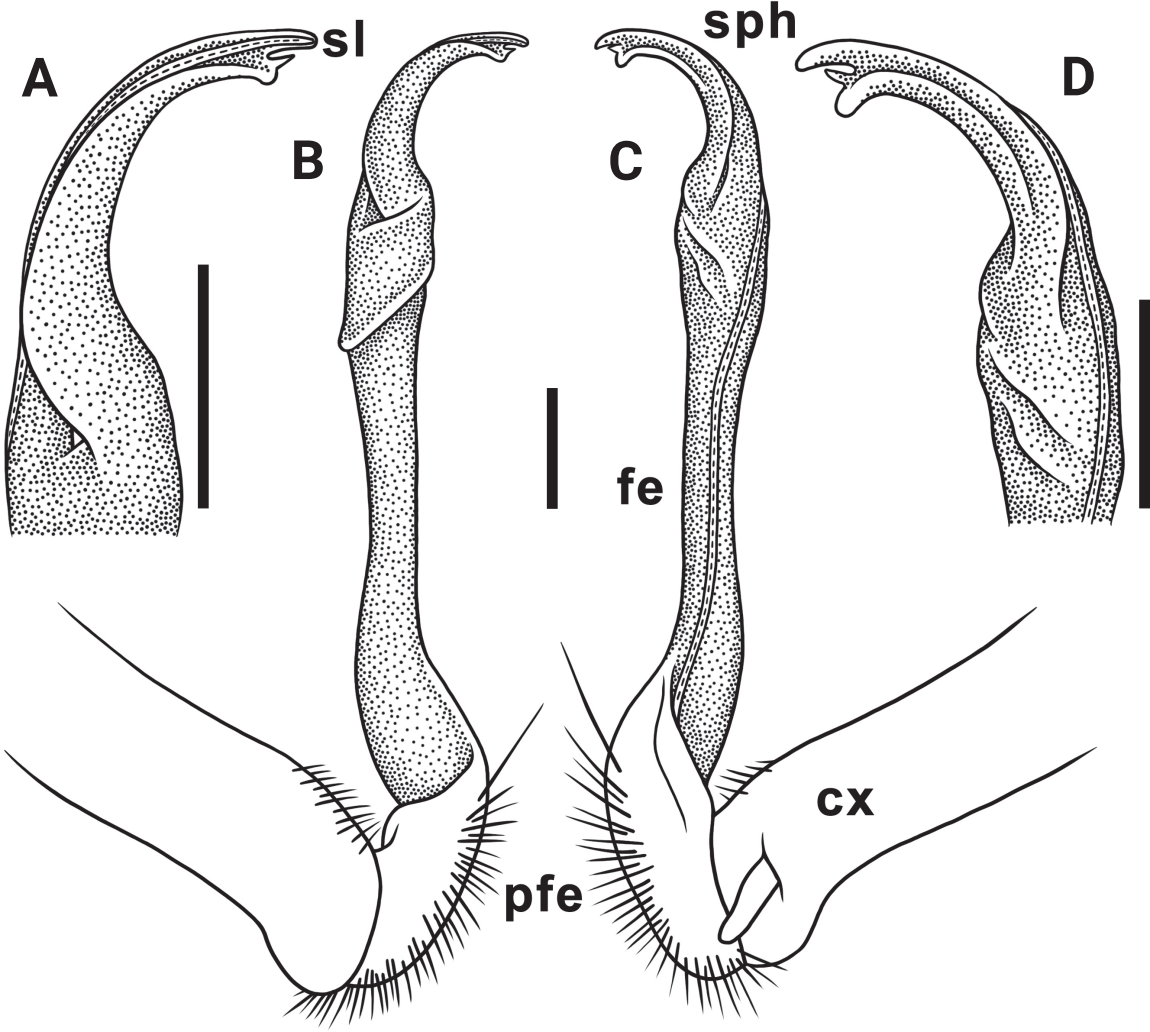
*Orthomorpha tergoaurantia* Likhitrakarn, sp. nov., ♂ holotype, right gonopod. A, D. Distal part of gonopod, sublateral and submesal views, respectively; B, C. Lateral and mesal views, respectively. Abbreviation: cx coxite, fe femoral part, pfe prefemoral part, sl solenomere, sph solenophore. Scale bars: 0.2 mm.

**Figure 4.**
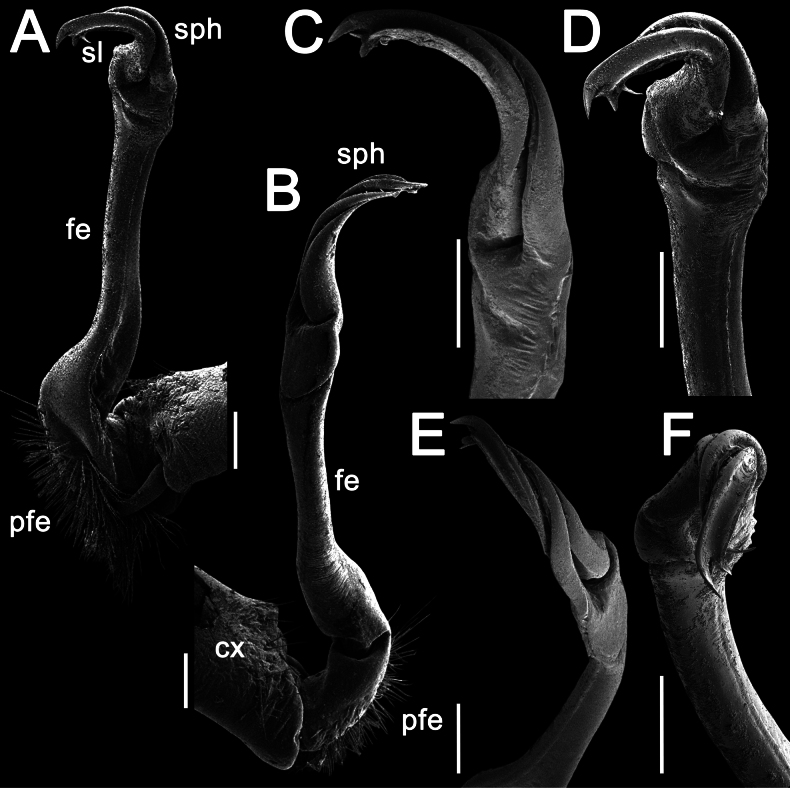
*Orthomorpha tergoaurantia* Likhitrakarn, sp. nov., ♂ holotype, right gonopod. A, B. Right gonopod, submesal and sublateral views, respectively; C–F. Distal part of right gonopod, mesal, submesal, subcaudal and suboral views, respectively. Abbreviation: cx coxite, fe femoral part, pfe prefemoral part, sl solenomere, sph solenophore. Scale bars: 0.2 mm.

Gonopods long, slender and suberect (Figs 3, 4). Coxite long and slender, slightly curved caudally, rather densely setose distodorsally (Figs 3B, C, 4A, B). Prefemoral part (pfe) densely setose, as usual, about 1/3 as long as acropodite (femoral + postfemoral parts) (Fig. 3B, C). Femoral part (fe) long and slender, slightly curved and suberect distad, with a postfemoral part demarcated by an oblique lateral sulcus (Figs 3B, 4B). Solenophore (sph) trifid, its terminal lobule longest, middle prong spiniform and shorter than subterminal lobule (Figs 3, 4A, C–F); solenomere (sl) long and flagelliform.

### ﻿ Orthomorpha efefai

Taxon classificationAnimaliaPolydesmidaParadoxosomatidae

Likhitrakarn sp. nov.

219CCE65-6B4F-51F4-8150-7E964467422A

https://zoobank.org/8B6A28C7-E961-4740-90DB-870FC6426686

Figs 5A, B, 6, 7, 8

#### Material examined.

***Holotype***: ♂ (CUMZ-PD0035), Cambodia, Battambang Province, Banan District, Chheu Teal, outside Neang Romsay Sak Cave (locality code C101), ca 50 m a.s.l., 12°57'34"N, 103°06'32"E, 31.07.2024, leg. R. Srisonchai. ***Paratypes***: 1 ♀ (CUMZ-PD0035), same data, together with holotype. 1 ♂, 3 juveniles (CUMZ-PD0036), Cambodia, Battambang Province, South-West Battambang, Phnom Romsay Sak, cave, 25 m a.s.l., 12°57'28"N, 103°06'32"E, 19.07.2017, leg. P. Jäger and S. Münnich.

#### Etymology.

The species name ‘efefai’ is a phonetic spelling representation of the Fauna and Flora Cambodia (formerly known by the acronym FFI). This new species is named in honour of their passionate commitment to protecting Earth’s biodiversity and acknowledges FFI Cambodia for supporting our team to explore the rich biodiversity of the country. When pronounced, ‘efefai’ is with the ‘e’ and ‘a’ being silent.

#### Diagnosis.

This new species seems to be particularly similar to *Orthomorpha parasericata* Likhitrakarn, Golovatch & Panha, 2010, a species known only from Surat Thani and Phang Nga provinces in southern Thailand, in having likewise broad, contrastingly lighter-colored paraterga, coupled with a stout gonopod telopodite and a bifid solenophore. However, it clearly differs by its smaller body size (28.4–33 mm long and 4.2–4.9 mm wide in males, 32.5 mm long and 4.6 mm wide in females, *vs* 32–37 mm long and 4.8–5.0 mm wide in males, 34–37 mm long and 4.8–5.3 mm wide in females of *O. parasericata*), as well as by the shape of the solenophore apex. Additionally, tarsal brushes are present until ♂ legs of ring 9 in the new species, whereas in *O. parasericata*, they are present only until ♂ legs of ring 5.

*Orthomorpha efefai* sp. nov. can also be distinguished from the sympatric *O. tergoaurantia* sp. nov., with which it shares a superficially similar gonopod structure. The key differences lie in the somatic characters. The metaterga in *O. efefai* sp. nov. are distinctly rugulose-tuberculate, bearing two rows of setiferous cones, especially on the anterior body rings (Fig. 6A, B). In contrast, the metaterga of *O. tergoaurantia* are mostly smooth and leathery, with setiferous knobs being far less pronounced (Fig. 2A–F). The male tarsal brushes extend until the legs of ring 9 in *O. efefai* sp. nov., but are far more extensive in *O. tergoaurantia* sp. nov., persisting until the legs of ring 17. Additionally, the caudal tooth of the pleurosternal carinae is traceable until ring 16 in *O. efefai* sp. nov., but only until rings 7 or 8 in *O. tergoaurantia* sp. nov. (Fig. 2B, D). Although broadly similar, the solenophore tip in *O. efefai* sp. nov. is distinctly bifid (Figs 7, 8), while in *O. tergoaurantia* sp. nov. it is trifid, with a small but clear middle prong (Figs 3, 4A, C–F).

#### Description.

Length 28.4–31.8 mm (♂), 32.5 mm (♀), width of midbody pro- and metazona 2.6–2.7 and 4.2–4.3 mm (♂) or 3.3 and 4.6 mm (♀), respectively.

**Figure 5.**
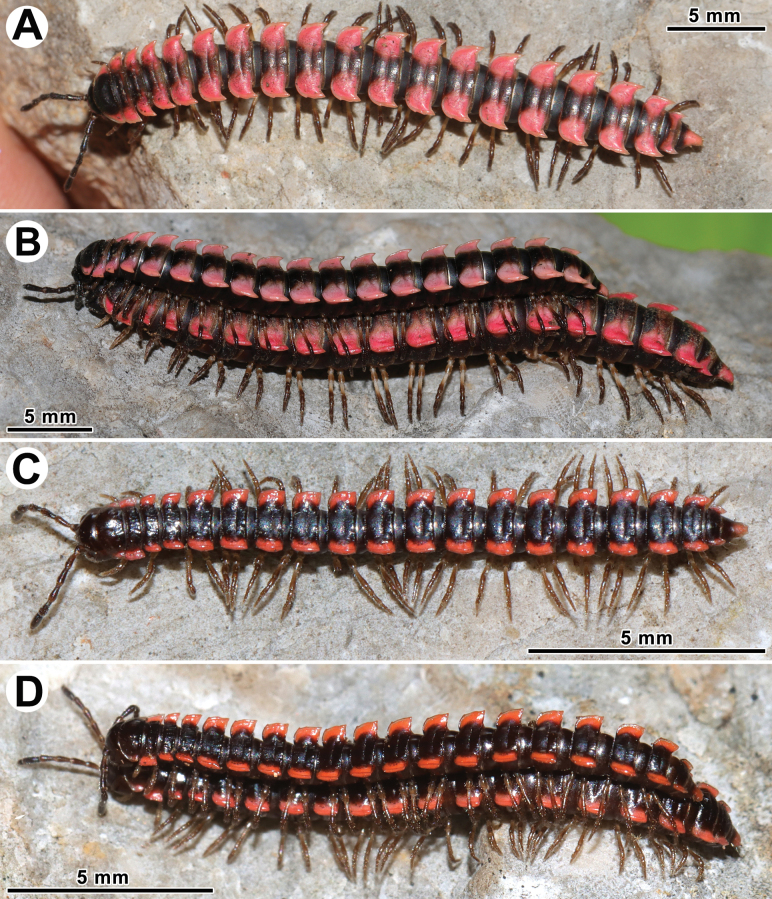
Habitus, live coloration. A, B. *Orthomorpha efefai* Likhitrakarn, sp. nov.; A. ♂ holotype; B. ♂ holotype (above) and ♀ paratype from Neang Romsay Sak Cave; C, D. *Orthomorpha battambangiensis* Likhitrakarn, sp. nov.; C. ♂ paratype; D. ♂ (above), ♀ paratypes from Phnom Kdoang Sampov.

**Figure 6.**
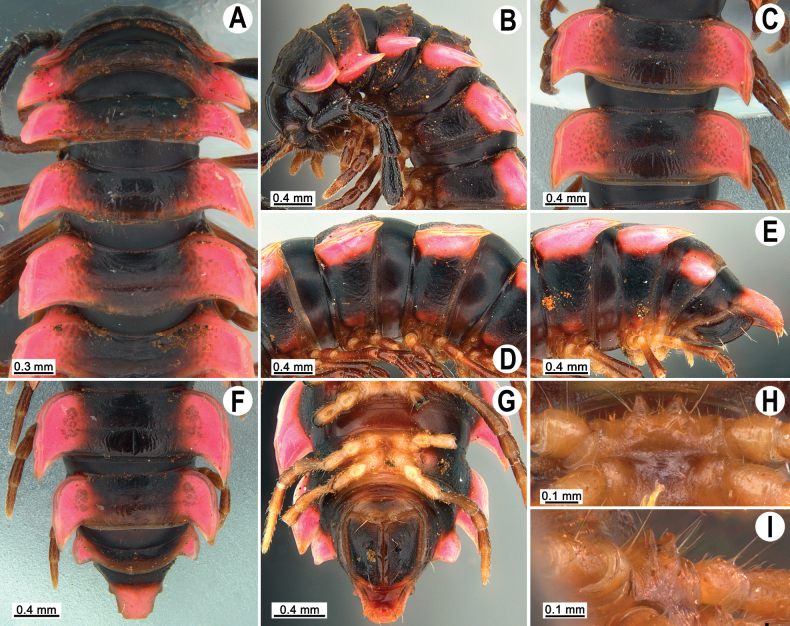
*Orthomorpha efefai* Likhitrakarn, sp. nov., ♂ holotype. A, B. Anterior part of body, dorsal and lateral views, respectively; C, D. Rings 10 and 11, dorsal and lateral views, respectively; E–G. Posterior part of body, lateral, dorsal and ventral views, respectively; H, I. Sternal cones between coxae 4, caudal and sublateral views, respectively.

Coloration of live animals blackish (Fig. 5A, B), with contrasting reddish pink or bright pink paraterga and epiproct, posterior halves of metaterga on rings 16–1 and antennae blackish, legs brown; coloration of alcohol material after 10 months of preservation faded to uniformly dark brown (Fig. 6A–G) with contrasting bright pink or pale pinkish paraterga and epiproct, legs brown to light grey-brown.

**Figure 7.**
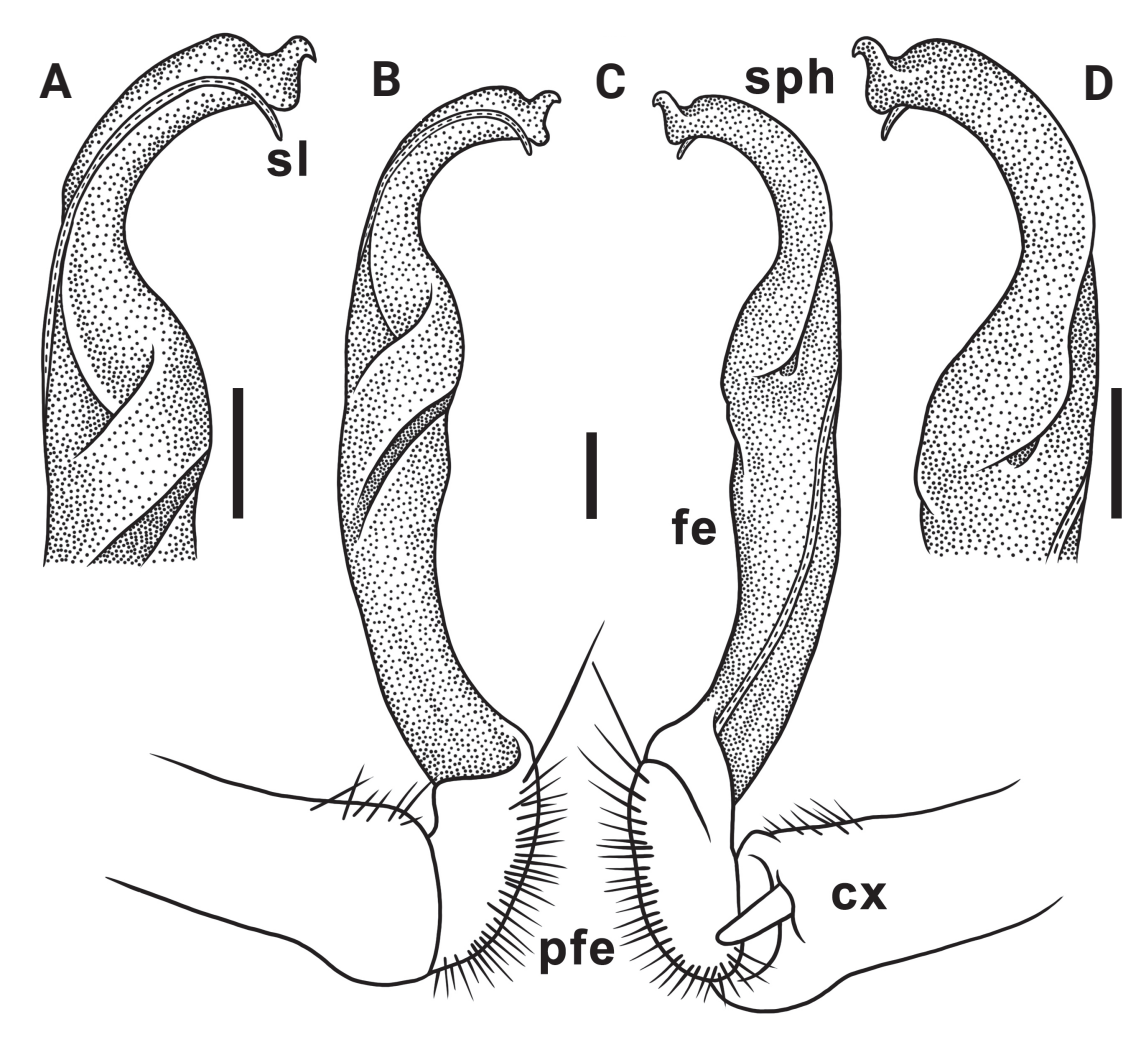
*Orthomorpha efefai* Likhitrakarn, sp. nov., ♂ holotype, right gonopod. A, D. Distal part of gonopod, sublateral and submesal views, respectively; B, C. Lateral and mesal views, respectively. Scale bars: 0.1 mm. Abbreviation: cx coxite, fe femoral part, pfe prefemoral part, sl solenomere, sph solenophore.

**Figure 8.**
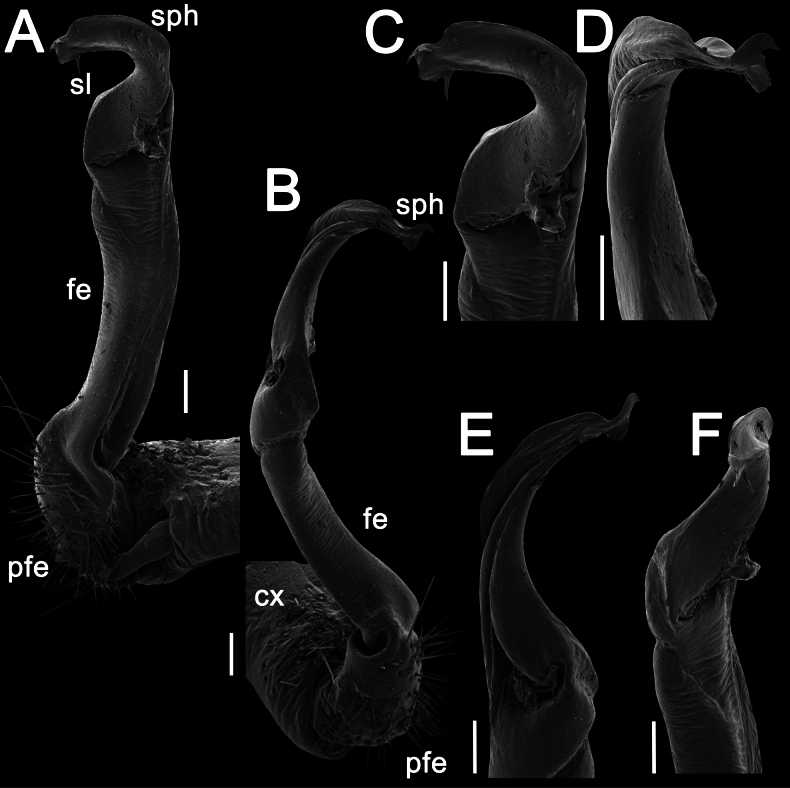
*Orthomorpha efefai* Likhitrakarn, sp. nov., ♂ holotype, right gonopod. A, B. Right gonopod, submesal and sublateral views, respectively; C–F. Distal part of right gonopod, submesal, sublateral, subcaudal and suboral, respectively. Abbreviation: cx coxite, fe femoral part, pfe prefemoral part, sl solenomere, sph solenophore. Scale bars: 0.1 mm.

Clypeolabral region and vertex sparsely setose, epicranial suture distinct. Antennae long (Fig. 5B), extending past ring 3 (♂) or reaching to ring 3 (♀) when stretched dorsally. In width, head < collum < ring 3 < 4 < 2 < 5 < 6 < 7–15 (Fig. 6A), thereafter body gently and gradually tapering. Collum with three transverse rows of strong setae: 3+3 anterior, 2+2 intermediate, and 3+3 posterior; a very faint incision laterally in posterior 1/3; caudal corner of paraterga pointed, dentiform, paraterga declined ventrad, not drawn past rear margin.

Tegument of metaterga shining, rugulose-tuberculate, especially so on several front metaterga; prozona very finely shagreened, metazona below paraterga faintly rugulose (Fig. 6A–F). Metaterga 2–5 with two rows of 2+2 anterior and 3+3 setiferous cones, usually slightly smaller cones in anterior (pre-sulcus) row and more evident ones laterally in posterior row (Fig. 6A, B); thereafter same pattern, but traceable only as insertion points in anterior row and as minute knobs gradually increasingly obliterate to become nearly absent from ring 10 onward. Tergal setae short, simple, slender, often abraded, about 1/4 metatergal length. Axial line traceable, especially clear on collum and following few metaterga. Paraterga very strongly developed (Fig. 6A–F), broad, all lying below dorsum (at about 1/4 body height), mostly subhorizontal, slightly upturned on rings 2–5, in lateral view moderately enlarged on pore-bearing rings, thinner on poreless ones (Fig. 6B, D, E); anterior margin well-developed, mostly nearly straight and narrowly bordered, fused to callus; caudal corner of most of paraterga very narrowly rounded, increasingly drawn past tergal margin, slightly curved mesad on rings 15–19 (Fig. 6B, D, E). Calluses on paraterga delimited by a sulcus only dorsally. Paraterga 2 broad, lateral edge with two small, but evident incisions, one in anterior 1/3, the other in posterior 1/3. Paraterga 3 and 4 each with an evident incision in anterior 1/3 at lateral margin (Fig. 6A). Lateral margins of following paraterga with two small incisions, one at midway, the other in posterior 1/3, the other at midway, caudal incision being smaller on pore-bearing rings (Fig. 6A, C, F). Posterior margin of paraterga concave, especially clearly so in rings 15–19. Ozopores evident, lateral, lying in an ovoid groove at about 1/3 metatergal length in front of posterior edge of metaterga (Fig. 6B, D, E). Transverse sulcus distinct (Fig. 6A–F), slightly incomplete on ring 2, complete and clearly visible on metaterga 3–18, shallow, reaching the bases of paraterga, arcuate, faintly beaded at bottom. Stricture between pro- and metazona rather wide, deep, beaded at bottom down to base of paraterga (Fig. 6A, C, F). Pleurosternal carinae complete crests on rings 2–4, following rings 5–7(8) each broken into an anterior bulge and a sharp caudal tooth, the latter gradually reduced in size to a small tooth until ring 16, thereafter missing (♂, ♀) (Fig. 6B, C, E).

Epiproct (Fig. 6E–G) conical, flattened dorsoventrally, subtruncate, with two evident apical papillae directed ventrocaudally (Fig. 6E, G); pre-apical papillae small, but evident, lying close to tip. Hypoproct subtrapeziform (Fig. 6G), 1+1 small setiferous knobs at caudal edge well-separated and evident.

Sterna sparsely setose, without modifications; cross-impressions rather deep; a paramedian pair of evident, fully separated, setose cones between ♂ coxae 4 (Fig. 6H, I). A conspicuous ridge in front of gonopod aperture. Legs moderately long and slender, midbody ones ca 1.0–1.2 (♂) (Fig. 5B) or 0.9–1.1 times (♀) as long as body height, prefemora without modifications, ♂ tarsal brushes present until leg 9.

Gonopods simple and suberect (Figs 7, 8). Coxite (cx) long and slender, slightly curved caudally, rather densely setose distodorsally (Figs 7B, C, 8A, B). Prefemoral part (pfe) densely setose, as usual, less than half the length of acropodite (femoral + postfemoral parts) (Fig. 7B, C). Femoral part (fe) slender, slightly curved and slightly enlarged distad, with a postfemoral part demarcated by an oblique lateral sulcus (Figs 7A, B, 8B, E). Solenophore (sph) with an evidently bifid tip, with a subterminal lobule (Figs 7, 8A–E) and a spiniform apical lobule (Figs 7, 8A–F); solenomere (sl) long and flagelliform (Figs 7A, B, 8A, C, E).

### ﻿ Orthomorpha battambangiensis

Taxon classificationAnimaliaPolydesmidaParadoxosomatidae

Likhitrakarn sp. nov.

7F18A5B1-4814-5124-A6B0-EA4154F90ABB

https://zoobank.org/C986E03C-AA55-4C48-ACFB-2087DB6460E5

**Figure 9.**
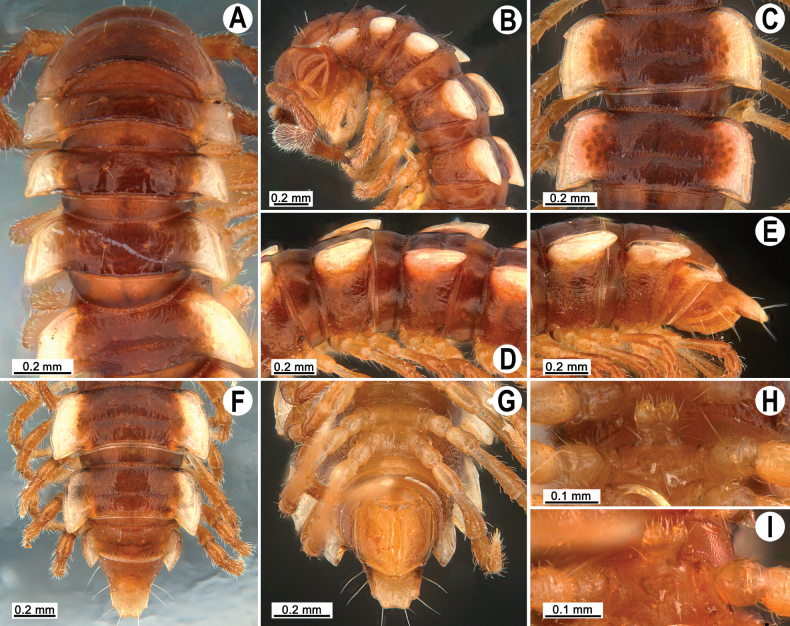
*Orthomorpha battambangiensis* Likhitrakarn, sp. nov., ♂ holotype. A, B. Anterior part of body, dorsal and lateral views, respectively; C, D. Rings 10 and 11, dorsal and lateral views, respectively; E–G. Posterior part of body, lateral, dorsal and ventral views, respectively; H, I. Sternal cones between coxae 4, subcaudal and sublateral views, respectively.

**Figure 10.**
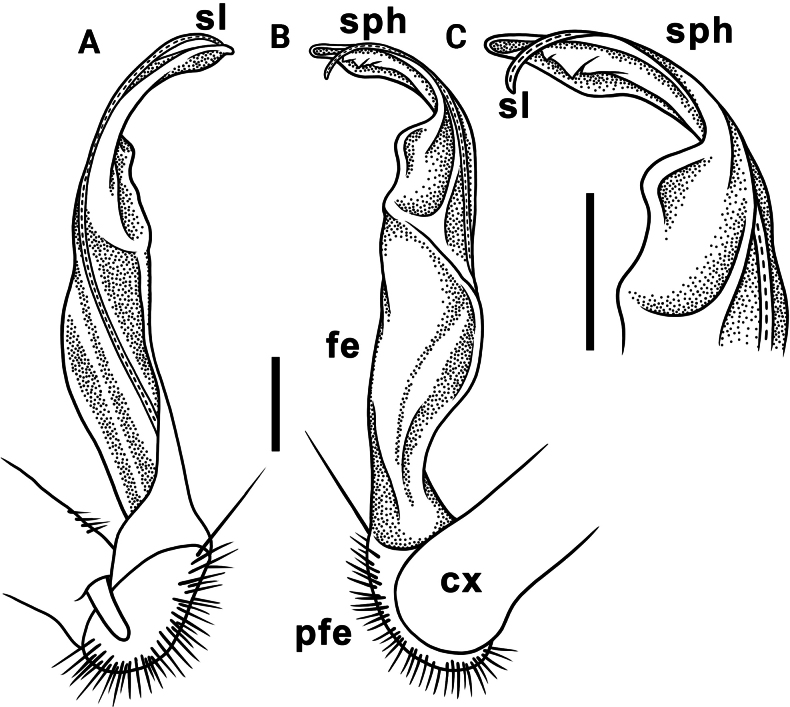
*Orthomorpha battambangiensis* Likhitrakarn, sp. nov., ♂ holotype, left gonopod. A, B. Mesal and lateral views, respectively; C. Tip of gonopod, mesal view. Abbreviation: cx coxite, fe femoral part, pfe prefemoral part, sl solenomere, sph solenophore. Scale bars: 0.1 mm.

**Figure 11.**
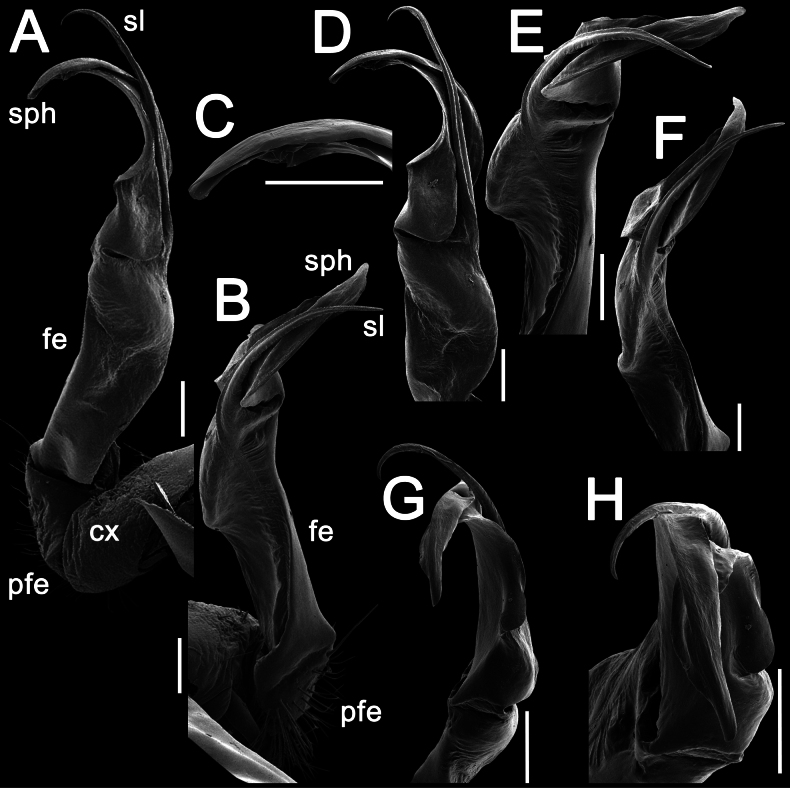
*Orthomorpha battambangiensis* Likhitrakarn, sp. nov., ♂ holotype, left gonopod. A, B. Right gonopod, sublateral and submesal views, respectively; C, H. Tip of gonopod, lateral and suboral views, respectively; D–G. Distal part of left gonopod, sublateral, submesal, subcaudal and suboral, respectively. Abbreviation: cx coxite, fe femoral part, pfe prefemoral part, sl solenomere, sph solenophore. Scale bar: 0.1 mm.

Figs 5C, D, 9, 10, 11

#### Material examined.

***Holotype***: ♂ (CUMZ-PD0037), Cambodia, Battambang Province, Banan District, Phnom Sampov, Killing Cave (locality code C098), ca 100 m a.s.l., 13°01'21"N, 103°05'55"E, 30.07.2024, leg. R. Srisonchai. ***Paratypes***: 5 ♂♂, 5 ♀♀ (CUMZ-PD0037), same data, together with holotype. 1 ♀ (CUMZ-PD0037), same District, Phnom Sampov, Kdoang Mountain (locality code C099), ca 120 m a.s.l., 13°00'53"N, 103°05'48"E, 30.07.2024, leg. R. Srisonchai.

#### Etymology.

The species epithet is an adjective formed from the type locality, Battambang Province, Cambodia.

#### Diagnosis.

This new species is distinguished by its notably small body size, being the smallest known in the genus *Orthomorpha*, measuring 12.4–14.6 mm (♂) or 17.2–17.8 mm (♀) in length, and 1.6–1.8 mm (♂) or 2.1–2.3 mm (♀) in width at midbody. The gonopod solenophore is complex, best described as trifid, bearing a small median denticle between the long terminal lobe and a smaller subterminal lobule. Additionally, tarsal brushes are present until ♂ legs of ring 9.

#### Description.

Length 12.4–14.6 mm (♂), 17.2–17.8 mm (♀), width of midbody pro- and metazona 1.0–1.1 and 1.6–1.8 mm (♂) or 1.5–1.7 and 2.1–2.3 mm (♀), respectively.

Coloration of live animals dark castaneous brown (Fig. 5C, D), with contrasting pale red or bright orange paraterga and epiproct; antennae dark brownish, venter and legs dark brown to brown; coloration of alcohol material after 10 months of preservation faded to uniformly reddish brown (Fig. 9A–G) with contrasting light yellow paraterga and epiproct; antennae, venter and legs brown to yellowish brown.

Clypeolabral region and vertex sparsely setose, epicranial suture distinct. Antennae long (Fig. 9B), reaching to ring 3 (♂, ♀) when stretched dorsally. In width, collum < head < ring 3 < 4 < 2 < 5 < 6 < 7–16 (Fig. 9A, C, F), thereafter body gently and gradually tapering. Collum with three transverse rows of strong setae: 4+4 anterior, 2+2 intermediate, and 3+3 posterior; a small incision laterally in posterior 1/3; caudal corner of paraterga pointed and dentiform; paraterga declined ventrad, not drawn past rear margin.

Tegument generally smooth and shining (Fig. 9A–F), metaterga only at places faintly rugulose, slightly more so near rear margin; prozona very finely shagreened, metazona below paraterga faintly rugulose (Fig. 9B, D, E). Postcollum metaterga with two transverse rows of setae: 2+2, mostly abraded setae in anterior (pre-sulcus) row, 3+3 in posterior (post-sulcus) one, the latter setae borne on minute tubercles, gradually reduced in size thereafter. Tergal setae simple, rather long, about 1/3 metatergal length, mostly abraded. Axial line traceable both on pro- and metazona. Paraterga strongly developed (Fig. 9A–F), lying level to or slightly above dorsum, only on rings 1–4, 18 and 19 either lying clearly below dorsum (♂) or all lying slightly below dorsum, set at about half of midbody height, subhorizontal (♀), in lateral view moderately enlarged in pore-bearing rings, thinner in poreless ones (Fig. 9B, D, E); shoulders broadly rounded, narrowly bordered, fused to callus; caudal corner narrowly rounded to pointed, slightly drawn past rear tergal margin (Fig. 9B, D, E). Calluses delimited by a sulcus only dorsally. Paraterga 2 broad, anterior edge convex, lateral edge with two acute denticles, one in anterior 1/3, the other at midway (Fig. 9A). Each following poreless ring with two incisions, each pore-bearing one with one, often evident incision in front of ozopore. Posterior edge of paraterga oblique, especially clearly so in rings 17–19. Ozopores evident, lateral, lying in an ovoid groove at about 1/3 metatergal length in front of posterior edge of metaterga (Fig. 9D, E). Transverse sulcus distinct (Fig. 9A, C, F), slightly incomplete on ring 19, complete and clearly visible on metaterga 5–18, rather deep, reaching the bases of paraterga, line-shaped, beaded at bottom. Stricture between pro- and metazona rather wide, shallow, beaded at bottom down to base of paraterga (Fig. 9A, C, F). Pleurosternal carinae well-developed, as complete, arcuate ridges with distinct caudal denticle on rings 2–8 (♂) (Fig. 9B, D) or rings 2–4 (♀), as a sharp caudal tooth, the latter gradually reduced in size down to a small tooth until ring 17 (♂), or broken into an anterior bulge and a caudal tooth on rings 5–8, retained only as a small, caudal, mostly rounded tooth on rings 9–16 (♀), entirely absent thereafter.

Epiproct (Fig. 9E–G) conical, flattened dorsoventrally, with two evident apical papillae; tip subtruncate; pre-apical papillae small, but visible, lying rather close to tip (Fig. 9F, G). Hypoproct nearly semi-circular, 1+1 setiferous knobs at caudal edge well-separated and evident (Fig. 9G).

Sterna sparsely setose, without modifications; cross-impressions shallow; with a paramedian pair of evident, anteroventrally directed, rounded prongs between ♂ coxae 4 (Fig. 9H, I). A conspicuous ridge in front of gonopod aperture. Legs long and slender, midbody ones ca 1.3–1.5 (♂) or 1.1–1.3 times (♀) as long as body height (Fig. 9B, F), prefemora without modifications, ♂ tarsal brushes present until leg 4.

Gonopods stout and suberect (Figs 10, 11). Coxite (cx) rather short, slightly curved caudally, rather densely setose distodorsally (Figs 10A, B, 11A, B). Prefemoral part (pfe) densely setose, less than half the length of acropodite (femoral + postfemoral parts) (Fig. 10A, B). Femoral part (fe) rather stout, slightly curved and faintly enlarged distad, with a postfemoral part demarcated by an oblique lateral sulcus (Figs 10A, B, 11A, B). Tip of solenophore (sph) faintly bifid (Figs 10B, C, 11A, C, D), with a subtruncate and long terminal lobe (Figs 10, 11A–H) and a small subterminal lobule (Figs 10B, C, 11A, C, D); solenomere (sl) long and flagelliform (Figs 10, 11A, B, D–H).

### ﻿Key to the species *Orthomorpha* known to occur in Cambodia, based on male characters (Likhitrakarn et al. 2011)

**Table:** 

1	Gonopod tip as a single, very small, rounded lobule. No evident modifications between ♂ coxae 4. Pantropical	***O. coarctata* (de Saussure, 1860)**
–	Gonopod tip bi- or trifid (Figs 3, 4, 6, 7, 9, 10). Modifications between ♂ coxae 4 mostly present (Figs 2H, I, 5H, I, 9H, I). Southeast Asia	**2**
2	Body smaller: 12.4–14.6 (♂) or 17.2–17.8 mm (♀) long and 1.6–1.8 (♂) or 2.1–2.3 mm (♀) wide. Sternal cones between ♂ coxae 4 fused basally into a single lamina (Fig. 9H, I). Tarsal brushes present until ♂ legs 4	***O* . *battambangiensis* Likhitrakarn, sp. nov.**
–	Body larger: 17–39.4 (♂) or 19–39.4 mm (♀) long and 2.0–4.9 (♂) or 2.0–5.7 mm (♀) wide. Sternal cones between ♂coxae 4 isolated (Figs 2H, I, 5H, I). Tarsal brushes present at least until ♂ legs 5 or extending further posteriorly	**3**
3	Pleurosternal carinae complete crests on rings 2–4, following rings 5–7(8) each broken into an anterior bulge and a sharp caudal tooth (Fig. 5B), thereafter increasingly strongly reduced until ring 16 (♂, ♀) (Fig. 5D, E). Legs shorter, midbody ones 1.0–1.2 (♂) or 0.9–1.1 times (♀) as long as body height. Gonopod telopodite stout and tip evidently bifid (Figs 6, 7)	***O* . *efefai* Likhitrakarn, sp. nov.**
–	Pleurosternal carinae complete crests on rings 2–4(3) (♂, ♀) (Fig. 2B), each with an evident, small, sharp denticle caudally (Fig. 2D, E). Legs longer, midbody ones ca 1.2–1.5 (♂) or 0.8–1.3 times (♀) as long as body height. Gonopod telopodite slender and tip trifid (Figs 3, 4)	**4**
4	Caudal corners of midbody paraterga moderately produced past tergal margin. Sternal cones between ♂ coxae 4 very small, nearly imperceptible, widely separated. Tarsal brushes present until ♂ legs 5	***O. hydrobiologica* Attems, 1930**
–	Caudal corners of midbody paraterga strongly produced past tergal margin (Fig. 2A–G). Sternal cones between ♂ coxae 4 mostly evident, higher and clearly separated (Fig. 2H, I). Tarsal brushes present at least until ♂ legs 7 or extending further posteriorly	**5**
5	Body smaller: 17–30 (♂) or 19–29 mm (♀) long and 2.0–3.1 (♂) or 2.0–3.4 mm (♀) wide. Caudal denticle of pleurosternal carinae traceable until ring 17 (♂) or 16 (♀). Tarsal brushes present until ♂ legs 7	***O. cambodjana* (Attems, 1953)**
–	Body larger: 35.4–39.4 (♂) or 35.4–39.4 mm (♀) long and 4.1–4.9 (♂) or 4.2–4.3 mm (♀) wide. Caudal denticle of pleurosternal carinae traceable until ring 7 or 8 (♂, ♀) (Fig. 2B, D). Tarsal brushes present until ♂ legs of ring 17	***O* . *tergoaurantia* Likhitrakarn, sp. nov.**

## ﻿Discussion and conclusions

This study provides a taxonomic review of the millipede genus *Orthomorpha* in Cambodia, resulting in the description of three new species. These results increase the known Cambodian *Orthomorpha* fauna to six species, while the genus currently has a total of 59 recognized species. With these additions, the diversity of this genus in Cambodia (6 species) is now comparable to the faunas of Myanmar (5 species) and Vietnam (8 species), though still lower than in Laos (9 species) and considerably more restricted than in Thailand (26 species) (Likhitrakarn et al. 2010, 2011, 2014a, 2019; Nguyen et al. 2025)

The Cambodian diplopod fauna currently totals 26 (Likhitrakarn et al. 2015, 2020; Srisonchai et al. 2020, 2023), this estimate being quite low compared to those of the neighboring countries: Thailand with 263 species (Likhitrakarn et al. 2023), Laos with 34 species (Likhitrakarn et al. 2014b), Vietnam with 280 species (Nguyen et al. 2025), and Myanmar with 92 species (Likhitrakarn et al. 2017). These numbers highlight the insufficient exploration efforts and the comparatively inadequate knowledge of Cambodian diplopod diversity.

**Figure 12.**
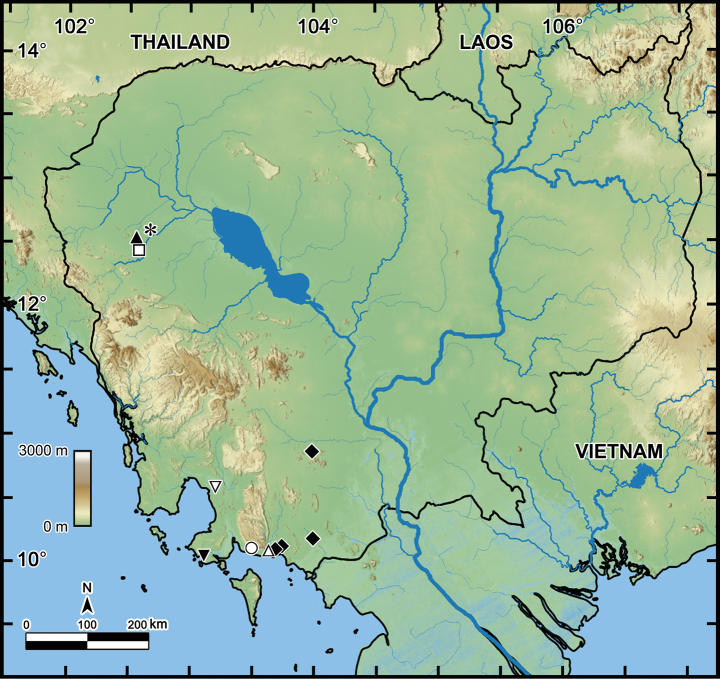
Distribution of *Orthomorpha* species in Cambodia (6 species). Asterisk: *Orthomorpha coarctata* (de Saussure, 1860); filled triangle: *Orthomorpha battambangiensis* Likhitrakarn, sp. nov.; open square: *Orthomorpha efefai* Likhitrakarn, sp. nov.; filled diamond: *Orthomorpha tergoaurantia* Likhitrakarn, sp. nov.; inverted open triangle: *Orthomorpha coarctata* (de Saussure, 1860) and *Orthomorpha cambodjana* (Attems, 1953); open triangle: *Orthomorpha cambodjana* (Attems, 1953); open circle: *Orthomorpha hydrobiologica* Attems, 1930; inverted filled triangle: *Orthomorpha cambodjana* (Attems, 1953) and *Orthomorpha hydrobiologica* Attems, 1930.

The distribution map (Fig. 12) reveals that the areas documented in this study are all geographically confined, primarily to the southern and western regions of Cambodia. The northern, central, and eastern regions of Cambodia have not yet been surveyed. These regions are ecologically diverse and may harbor a variety of undiscovered millipede species.

Considering Cambodia’s advantageous geographic position within the Indo-Burmese biodiversity hotspot, an area characterized by exceptional species richness and high levels of endemism across various taxa (ADB 2000), more thorough future investigations are expected to significantly enhance our comprehension of *Orthomorpha* and, more generally, Diplopoda diversity. The outstanding variety and endemism shown by several taxonomic groups in Cambodia highlight the need for continued study and conservation initiatives. For example, the genus *Tylopus* Jeekel, 1968, previously unrecorded in Cambodia, has recently been shown to contain at least two endemic species in the country’s montane forests (Srisonchai et al. 2023). Similarly, our current work on *Orthomorpha* has doubled the number of known genus’ members in the country, with all three newly described species appearing to be endemics. These discoveries in large-bodied, conspicuous genera, alongside findings in smaller, more cryptic groups like the micropolydesmoid genus *Eutrichodesmus* Silvestri, 1910 (Srisonchai et al. 2020), underscore the potential for uncovering significant, unique biodiversity with further dedicated exploration.

## Supplementary Material

XML Treatment for Orthomorpha

XML Treatment for Orthomorpha hydrobiologica

XML Treatment for Orthomorpha cambodjana

XML Treatment for Orthomorpha tergoaurantia

XML Treatment for Orthomorpha efefai

XML Treatment for Orthomorpha battambangiensis

## ﻿Additional information

### Use of AI

No use of AI was reported.

### Author ORCIDs

Natdanai Likhitrakarn https://orcid.org/0000-0002-1306-317X

Chhin Sophea https://orcid.org/0000-0002-5612-7532

Pablo Sinovas https://orcid.org/0000-0001-5396-8910

Ekgachai Jeratthitikul https://orcid.org/0000-0002-3477-9548

Arthit Pholyotha https://orcid.org/0000-0001-6677-1164

Ruttapon Srisonchai https://orcid.org/0000-0002-7142-0999

Chirasak Sutcharit https://orcid.org/0000-0001-7670-9540

### Data availability

All of the data that support the findings of this study are available in the main text.
